# Tattoo removal in the typical adolescent

**DOI:** 10.1186/1756-0500-4-209

**Published:** 2011-06-21

**Authors:** Luca Cegolon, Vincenzo Baldo, Carla Xodo, Francesco Mazzoleni, Giuseppe Mastrangelo

**Affiliations:** 1 Department of Environmental Medicine and Public Health, Padua University, Padua, Italy; 2 Imperial College London, School of Public Health, London, UK; 3 Department of Educational Sciences, Padua University, Padua, Italy; 4 Department of Medical and Surgical Sciences, Plastic Surgery Unit, Padua University Hospital, Padua, Italy

**Keywords:** survey, epidemiology, tattoo removal, piercing, secondary school adolescents, health education

## Abstract

**Background:**

Although popular tattoos are often regretted later on for different reasons. Nevertheless, tattoo removal is a complicated and costly procedure seldom providing satisfactory results. The aim of this study was to investigate the awareness of the implications of tattoo removal among a substantial sample of Italian secondary school adolescents.

**Findings:**

Students were recruited by a stratified convenience sample and surveyed by a self administered questionnaire. Logistic regression analysis was performed, reporting adjusted Odds Ratios (OR), with 95% Confidence Interval (CI).

4,277 pupils returned a usable questionnaire. Piercings were more frequently undertaken than tattoos. Only 40% of the respondents were aware of the issues related to tattoo removal. Males and pupils with younger fathers were less likely to be aware, whereas students satisfied with their physical appearance and those with a positive attitude towards body art were more likely to be aware.

**Conclusions:**

Male adolescents with younger fathers can be regarded as the ideal target of corporate health education programs driven by school counsellors and primary care physicians.

## Findings

### Background

Although popular [1-3], tattoos are often regretted subsequently for different reasons: desire to improve physical appearance; loss of art value or uniqueness; end of a relationship; conformity and peer pressure; family pressure; severing ties with a previous stage of life; end of group/gang affiliation; increase employment chances; embarrassment, social rejection/stigma; or just to remove it without further reason provided [4-9].

Houghton [10] reported that 44% of interviewees regretted obtaining their tattoos, with 26% of them considering their removal. According to Saunders [11] the percentage of people requiring tattoo removal was 33%. Varma [6] and Dupront [12] reported that 28% of tattooed individuals regretted obtaining their tattoo barely one month after its application.

Besides being an established risk factor for severe/chronic systemic infections and localized dermatological disorders [13-15], tattoos are also difficult to remove. Despite advances in laser technology, removing a tattoo is a painstaking process, usually involving several treatments and considerable expense, and complete removal without scarring may be impossible [16,17]. In view of the above we chose to investigate a sizable sample of Italian secondary school adolescents and their awareness about tattoo removal. This issue had never previously been studied in this age group. This is a secondary analysis partially using some data already published [3,18].

## Methods

### Sample strategy

In each of the seven provinces of the Veneto Region, Northeastern Italy, six schools (belonging to each of the six types of Italian public secondary schools) were chosen by convenience sampling, on the basis of individual negotiations with the respective schools' head teachers. The original sample included 42 (= 6 × 7) schools, 41 of which eventually agreed to take part. Two sections of pupils attending the 1^st^,3^rd^, 5^th ^school years were randomly selected in each school. The 4,524 students attending these classes (adolescents 13-21 years old) comprised the study cohort.

### Data collection

The field survey was undertaken in 2007. In each classroom a researcher explained the purpose and methods of the study before providing the sample with an anonymous 22 item self administered questionnaire. Students were free to refuse to take part in the survey and were assured that their responses would remain confidential. The time necessary to complete the questionnaire was 10 minutes, and the questionnaire was returned in a sealed envelope after its completion.

The multiple choice questionnaire collected information on the outcome and independent variables, mainly socio-demographic information, that were found to be significant predictors of body art attitudes in other westernized countries [1,2,4,10,15,19,20]. We felt that this was particularly the case in our sample, mainly composed of underage students, hence likely to be living at home with the respective family of origin.

The former was a binary variable which was 1 if the student answered: "*I am aware it would be difficult (to remove) *" or "*I do not think I will try to remove it*" (and 0 otherwise) to the question: "*Have you considered the difficulties involved in the removal of tattoos?*". The independent variables were: place of residence ("city" any Province capital, "town" >15,000 inhabitants; "small town" <15,000 inhabitants); Province of residence; single parent household; number of siblings; sex and age of each sibling; father's/mother's age; education level of father/mother (low = junior secondary school, corresponding in Italy to attending school until 16 years of age; medium = secondary school; high = University or postgraduate degree); satisfaction with physical appearance (yes; fairly; no); attitude towards tattooing and piercing separately (indifferent or not interested; interested or keen to try; already experienced). Students declaring to be indifferent/not interested or interested/keen to try had never experienced piercing/tattoo before. Students who already had a piercing/tattoo included those who had removed it. Furthermore the questionnaire also collected information on the application of piercings and tattoos by body area (head, legs, arms, torso, abdomen) and the number of them removed.

The response rate was 100%, but after cleaning the dataset the observations were reduced to 4,277, 95% ( = 4,277/4,524) of the original sample.

### Statistical analysis

Weighted multivariable logistic regression analysis was employed, reporting Odds Ratios (ORs) of differences between the groups compared; ORs were weighted for gender and age using 2007 census data to make the results more representative of the adolescents of the Veneto Region aged 13 to 21 years. The explanatory variables of the final model were selected from an initial list of independent variables (see above: Data Collection) by backward stepwise logistic regression analysis using p <0.05 as a criterion. Missing values were excluded, and complete case analysis was performed.

Stata 11 software (Stata Corporation, College Station, Texas, USA) was used for the statistical analysis.

## Results

Table 1 shows that strata were of roughly similar size concerning age, school year attended, Province of residence, age of mother and father, satisfaction with physical appearance. Most of the students were females, resided in small towns, in families with both parents, with more than two children and with a low or medium level of socioeconomic status, as shown by the educational level of their respective parents. Prevalence of tattoo was 6%, whereas body piercing was 20%; 47% of the non-tattooed considered tattoo. The implications of tattoo removal were not sufficiently understood, as only 40% ( = 1,624/2,462) of the respondents seemed aware of the associated problems (data not shown).

**Table 1:** Frequency distribution of the outcome variable ("awareness of the implications of tattoo removal") by independent variables, among the 4,277 secondary school students

INDEPENDENT VARIABLES		No. (%)**	Outcome	
		
			Yes (%)*	No (%)*
Sex	Female	2,789 (65.2)	1,241 (46.5)	1,428 (53.5)
	
	Male	1,488(34.8)	383 (27.0)	1,034 (73.0)

Age	<16 years	1,494 (34.9)	523 (36.9)	895 (63.1)
	
	16-17 years	1,501 (35.1)	618 (43.0)	820 (57.0)
	
	18+ years	1,282 (30.0)	483 (39.3)	747 (60.74)

School year	1st	1,566 (36.6)	547 (36.8)	940 (63.2)
	
	3rd	1,478 (34.6)	609 (42.9)	811 (57.1)
	
	5th	1,181 (28.8)	468 (39.7)	711 (60.3)

Residence (missing: 179)	City centre	850 (20.7)	312 (38.3)	503 (61.7)
	
	City outskirt	979 (23.9)	382 (40.9)	553 (59.1)
	
	Town	412 (10.1)	145 (35.5)	252 (63.5)
	
	Small town	1,857 (45.3)	722(40.8)	1,049 (59.2)

Province of residence (missing: 32)	Belluno	509 (12.0)	180 (36.7)	310 (63.3)
	
	Verona	674 (15.9)	288 (44.4)	361 (55.6)
	
	Vicenza	402 (9.5)	170 (44.2)	216 (55.8)
	
	Padua	739 (17.4)	274 (38.5)	446 (61.5)
	
	Venice	554 (13.1)	196 (36.8)	346 (63.2)
	
	Treviso	621 (14.6)	234 (39.7)	375 (60.3)
	
	Rovigo	746 (17.6)	269 (38.6)	454 (61.4)

Satisfaction with physical appearance (missing: 69)	Yes	1,511 (35.9)	485 (33.5)	964 (66.5)
	
	Fairly	2,258 (53.7)	927 (42.7)	1,244 (57.3)
	
	No	439 (10.4)	199 (47.5)	220 (52.5)

Single parent household	No	3,806 (89.0)	1430 (39.4)	2,285 (60.6)
	
	Yes	471 (11.0)	194 (42.7)	266 (57.3)

No. of siblings (missing: 95)	0	779 (18.6)	294 (7.1)	448 (12.1)
	
	1	2,349 (56.2)	912 (19.8)	1,347 (37.1)
	
	2+	1,054 (25.2)	389 (7.9)	615 (16.1)

Senior sibling of same sex	No	3,963 (92.7)	1,535 (40.6)	2,250 (59.5)
	
	Yes	314 (7.3)	89 (29.6)	212 (70.4)

Father's age (missing: 409)	<49 years	1,824 (47.2)	691 (396)	1,056 (60.5)
	
	49+ years	2,044 (52.8)	807 (41.3)	1,149 (58.7)

Mother's age (missing: 337)	<47 years	2,058 (52.2)	799 (40.6)	1,168 (59.4)
	
	47+ years	1,882 (47.8)	724 (40.1)	1,082 (59.9)

Mother's education (missing: 144)	Low	1,456 (35.2)	585 (41.9)	812 (58.1)
	
	Medium	2,007 (48.6)	758 (39.4)	1,165 (60.6)
	
	High	670 (16.2)	238 (37.4)	399 (62.6)

Father's education (missing: 216)	Low	1,353 (33.3)	544 (41.9)	756 (58.2)
	
	Medium	1,917 (47.2)	739 (40.1)	1,103 (59.9)
	
	High	791 (19.5)	262 (35.2)	482 (64.8)

Attitude towards piercing (missing: 100)	Indifferent/Non interessted	2,276 (54.5)	580 (26.5)	1,607 (73.5)
	
	Interested/Keen to try	1,061(25.4)	560 (53.3)	491 (46.7)
	
	Done	840 (20.1)	478 (57.5)	353 (42.5)

Attitude towards tattoo (missing: 191)	Indifferent/Not interesested	1,900 (46.5)	380 (20.0)	1,520 (80.0)
	
	Interested/Keen to try	1,928 (47.2)	1,107 (57.4)	821 (42.6)
	
	Done	258 (6.3)	137 (53.1)	121 (46.9)

Yes = aware; No = not aware; Number (No.) and weighted percentage (%)*

* Row Percentage

** Column percentage

Boys were more likely to have a tattoo; among girls 48% ( = 1,358/2,789) were considering tattoos. Of those without any body modification 47% ( = 1,406/3,148) were interested in tattoos, and 32% ( = 1,005/3,148) in piercings. 66% ( = 558/840) of those with a piercing were underage, the equivalent for tattoo being 61% ( = 157/258). 166 individuals reported having both piercing and tattoo and 152 of these (92% = 152/166) were <18 years of age (data not shown).

Table 2 shows the weighted multivariable logistic regression model for the awareness of the difficulty related to tattoo removal. Males were less likely to be aware of the implications of tattoo removal, whereas students unsatisfied or fairly satisfied with their physical appearance, with older fathers, and those considering or already having a tattoo or a piercing were more likely to be aware. This model of multivariable logistic regression was fitted on 2,363 complete observations.

**Table 2:** Weighted multivariable logistic regression analysis: Odds Ratio (OR) and 95% Confidence Interval (CI)

EXPLANATORY VARIABLES		OR† (95%CI)
Sex	Female	*Reference*
	
	Male	0.53 (0.43; 0.66)

Satisfaction with physical appearance	Yes	*Reference*
	
	Fairly	1.39 (1.11; 1.73)
	
	No	1.25 (0.87; 1.78)

Attitude towards tattoo	Indifferent/Not interested	*Reference*
	
	Interested/Keen to try	4.65 (3.70; 5.84)
	
	Done	4.00 (2.63; 6.10)

Attitude towards piercing	Indifferent/Not interested	*Reference*
	
	Interested/Keen to try	1.76 (1.34; 2.26)
	
	Done	1.91 (1.42; 2.57)

Father's Age	< 49 years	*Reference*
	
	> 49+ years	1.26 (1.04; 1.54)

Multivariable regression model fitted on 2,363 complete observations

† ORs weighted for sex and age to make the results more representative of the adolescents of the Veneto Region aged 13-21 years

## Discussion

Decisions to remove a tattoo can be influenced by cost in terms of pain, money and risk of scarring [4], or simply ignorance of the availability of the service [6]. Patients regretted their tattoos for a median of 14 [10,12] or 10 years [4] before requesting removal, which could be due to awareness/concern regarding removal. In our study this awareness was higher among students already having or considered a tattoo or a piercing. Similarly, Houghton [10] found that the highest level of awareness of the health risks related to body art was among the group at greatest risk, boys with tattoos, suggesting that some males may be attracted to tattoos because of their known risks. However, as the human body changes over time and styles change with the seasons, the tattoo that seemed stylish at first may become dated, embarrassing and may be regretted later on.

Conversely in another study, students with a positive attitude towards body modifications (piercing and tattoo) were less aware of the health related risks [18].

Females and students with relatively older fathers were more likely to be aware of the issues surrounding tattoo removal in the present study. Based on these results anticipatory guidance and health education programs could be tailored to target students with younger fathers and males, also more likely to obtain a tattoo in this cohort (consistent with other reports) [2,19,20]. The educational level of either parents [21], especially the fathers' [3], has been found to be inversely correlated with body art attitude and should therefore be considered in the field of health promotion.

Future educational campaigns and guidance should also be aimed towards specific groups obtaining tattoos: underage people, women (presenting for tattoo removal more than men according to Armstrong [4]) and adolescents without body modifications. Another important aspect to be considered is the substantial percentage of adolescents desiring a tattoo (higher than that reported by Laumann [2]), who could achieve their aspiration once adults.

### Study limitations

A representative random sample design unfortunately was not feasible as the schools were recruited on the basis of individual agreements with the respective head teachers. Furthermore convenience sampling (which is also vastly employed in the current research on body art among adolescents [1,10,22,23]) ensured representation of all the 7 Provinces of the Region and each of the 6 types of Italian public secondary schools.

As education is currently compulsory until the age of 16 in Italy, our findings might not be generalizable to the older population or to drop outs/street youth, the latter reportedly being more likely to adopt risk taking behavior including body modification [24].

Our outcome "*awareness of the implications of tattoo removal*" was deliberately chosen to be not too specific. Indeed as 60% of our respondents were minors we aimed to investigate their knowledge/awareness at a general level. Furthermore this topic had never been investigated before and this was meant to be an exploratory study to possibly generate further hypotheses. Based on our findings we therefore believe further research is recommended in other westernized regions to investigate in more depth adolescents' awareness about specific aspects of tattoo removal such as the type of medical/surgical procedures available, their efficacy, their economic cost and their associated risks (especially pain and scarring).

## Conclusions

The present study has identified male adolescents, and especially those with younger fathers, as risk subgroups. This is important so that educational interventions may be targeted to those most at risk. School counselors and primary care physicians should therefore engage boys (rather than girls) and also younger (rather than older) fathers. This multi agency team should encourage and possibly dissuade children from impulsive tattooing, a practice often undertaken to comply with a social fashion, or because of peer pressure.

## Ethical approval

Not required. Approval for conducting the survey was obtained from the SSIS's review board and each school's head teacher.

## Competing interests

The authors declare that they have no competing interests.

## Authors' contributions

LC participated in the design and interpretation of the study, collected the data, performed the statistical analysis and drafted the paper, CX participated in the design of the study, the organization of the research team and is the guarantor, FM participated in the design of the study and the organization of the research team; GM contributed to the analysis, the interpretation of the study, and the drafting of the paper. VB participated in the design of the study, data collection and writing of the paper. The VAHP working group contributed to the survey and the data storage. All authors read and approved the final manuscript.
